# Phase I study of TQB3602, an oral proteasome inhibitor, in relapsed and refractory multiple myeloma

**DOI:** 10.1002/cam4.7435

**Published:** 2024-07-19

**Authors:** Wenjiao Tang, Yan Li, Li Zhang, Xushu Zhong, Qiushi Liang, Yuhuan Zheng, Yuzhang Liu, Yafei Wang, Xunqiang Wang, Yun Zeng, Baijun Fang, Li Zheng, Ting Niu

**Affiliations:** ^1^ Department of Hematology, West China Hospital Sichuan University Chengdu China; ^2^ Department of Hematology Henan Cancer Hospital Zhengzhou China; ^3^ Chia Tai Tianqing Pharmaceutical Group Co., LTD. Nanjing Jiangsu China; ^4^ Department of Hematology The First Affiliated Hospital of Kunming Medical University Kunming China; ^5^ Department of CTC Laboratory, West China Hospital Sichuan University Chengdu China

**Keywords:** antitumor activity, maximum tolerated dose, proteasome inhibitors, relapsed and refractory multiple myeloma

## Abstract

**Objective:**

TQB3602 is a novel orally bioavailable proteasome inhibitor. This study is the first‐in‐human phase I clinical trial to evaluate the safety, tolerability, pharmacokinetics, and preliminary efficacy of TQB3602 in relapsed/refractory multiple myeloma (RRMM).

**Methods:**

This is a multicenter phase I clinical trial consisting of the 3+3 dose‐escalation phase and dose expansion phase. Patients with MM who have received ≥2 prior antimyeloma therapies were enrolled. TQB3602 is administered at a dose of 0.5~7mg on days 1, 8, 15 in 28‐day cycle.

**Results:**

Twenty‐five RRMM patients who relapsed or failed ≥2 lines of therapies were enrolled in the dose escalation phase. Two patients in the 7.0 mg dose group developed dose‐limiting toxicity events (one with grade 2 peripheral neuropathy [PN] complicated by pain and one with diarrhea and abdominal pain), leading to a maximum tolerated dose of 6.0 mg. Any‐grade adverse events (AEs) occurred in 24 (96.0%) patients, while grade ≥3 AEs occurred in 13 (52.0%). The most common grade ≥3 AEs was anemia (6, 24.0%). The incidence rate of PN was 16% with no grade ≥3 PN occurred. TQB3602 was rapidly absorbed, resulting in a time‐to‐plasma peak concentration of 0.8–1.5 h. The mean half‐life was approximately 82 h. The AUC_last_ and *C*_max_ were approximately 1.9 times higher on day 15 than on day 1. Among 22 response‐evaluable patients, 63.7% achieved stable disease or better.

**Conclusions:**

TQB3602 is well tolerated, with a favorable neurotoxicity profile, and has shown preliminary efficacy in patients with RRMM. The anticipated therapeutic dose was 6 mg and was adopted for an ongoing dose‐expansion phase.

## 1 INTRODUCTION

The proteasome is a multicatalytic proteinase complex responsible for the degradation of various protein substrates within normal and transformed cells. The proteasome is a proven target in multiple myeloma (MM), including relapsed and refractory multiple myeloma (RRMM).^1^, ^2^ With the advancement of research, novel proteasome inhibitors such as ixazomib and carfilzomib have emerged, offering effective therapy alternatives for patients with RRMM.^3^ Proteasome inhibitors‐based therapies have led to more profound and more durable responses, resulting in significantly improved survival for RRMM, and are the current mainstay of MM therapy.^4^, ^5^

Ixazomib is the first boronate‐based third‐generation proteasome inhibitor approved for oral administration in patients with MM.^6^, ^7^, ^8^ It suppresses the chymotrypsin‐like activity of the β5 subunit of the 20S proteasome and is elective and reversible.^3^ Previous clinical trials demonstrated the antitumor effect and survival benefit of ixazomib‐containing regimens in RRMM.^9^, ^10^ Regarding safety, the most frequently reported adverse drug reactions were grade 1–2 fatigue, thrombocytopenia, nausea, and diarrhea,^11^ which were manageable and acceptable. Notwithstanding, some patients (any grade: 27%, grade 3: 2%) developed peripheral neuropathy,^11^ which could be caused by off‐target effects following ixazomib use.^12^ Thus, developing a novel proteasome inhibitor with a broader safety window by eliminating off‐target effects is necessary to enhance the treatment's efficacy and safety and improve the prognosis of patients with RRMM.

TQB3602 is a novel orally bioavailable proteasome inhibitor^13^ based on ixazomib and developed after multiple rounds of optimization. It incorporates an acridine ring into the structure of ixazomib, preserving its high activity and selectivity while improving metabolic stability. Comparable to ixazomib, TQB3602 is rapidly hydrolyzed to its active component, boric acid, in the form WXFL10410333, when it comes into contact with an aqueous solution under physiological circumstances. Preclinical efficacy studies have shown that TQB3602 has antitumor efficacy in the MM.1S MM model,^13^ and the in vitro and in vivo toxicological studies have also demonstrated that TQB3602 has similar toxic effects to ixazomib. A preclinical study demonstrated that TQB3602 displayed potent kinase inhibiting activity for 20S proteasome enzyme with IC_50_ 15.3 nM and inhibited cell proliferation in MM.1S multiple myeloma cell line with IC_50_ 10.1 nM. In MM.1S multiple myeloma xenograft models, TQB3602 showed a tumor growth inhibition rate >100% at 7 mpk and had a more evident effect of prolonging survival compared with ixazomib.^13^

Therefore, given the antitumor activity and favorable toxicological profile of TQB3602 in preclinical studies, we conducted a phase I clinical trial to explore the preliminary safety, tolerability, pharmacokinetics, and efficacy of TQB3602 in patients with RRMM.

## 2 METHODS

### 2.1 Study design and patients

This multicenter phase I clinical trial was conducted in China from May 2020 to October 2021. The inclusion criteria were (1) aged 18–75 years old, (2) patients with MM who received ≥2 prior antimyeloma therapies (including lenalidomide or thalidomide, bortezomib, and glucocorticoids) and received or were ineligible for hematopoietic stem cell transplantation (HSCT), (3) have at least one measurable lesion: serum M protein ≥5 g/L, or urine M protein ≥200 mg/24 h; patients with only serum‐free light‐chain as measurable lesions: serum‐free light chain level ≥10 mg/dL and abnormal kappa/lambda free light‐chain ratio, and (4) Eastern Cooperative Oncology Group (ECOG) performance status (PS) score 0–2, and (5) expected survival of more than 12 weeks. The exclusion criteria were (1) patients with ≥grade 2 peripheral neuropathy or grade 1 peripheral neuropathy with pain, (2) received chemotherapy, radiotherapy, targeted therapy, immunotherapy, or other systemic antitumor therapy within 14 days before the first dose of TQB3602, (3) previous allogeneic‐HSCT, (4) received autologous‐HSCT within 12 weeks before enrollment, or (5) received ixazomib less than five half‐lives before the first dose of TQB3602. The detailed inclusion and exclusion criteria are presented in Table S1.

This study was approved by the Ethics Review Board of West China Hospital, Sichuan University (2019 clinical trial [Western Medicine] approval [243]). In accordance with the Declaration of Helsinki, written informed consent was obtained from all participants before enrollment. The trial was registered with ClinicalTrials.gov (NCT04275583).

### 2.2 Procedure

**FIGURE 1:**
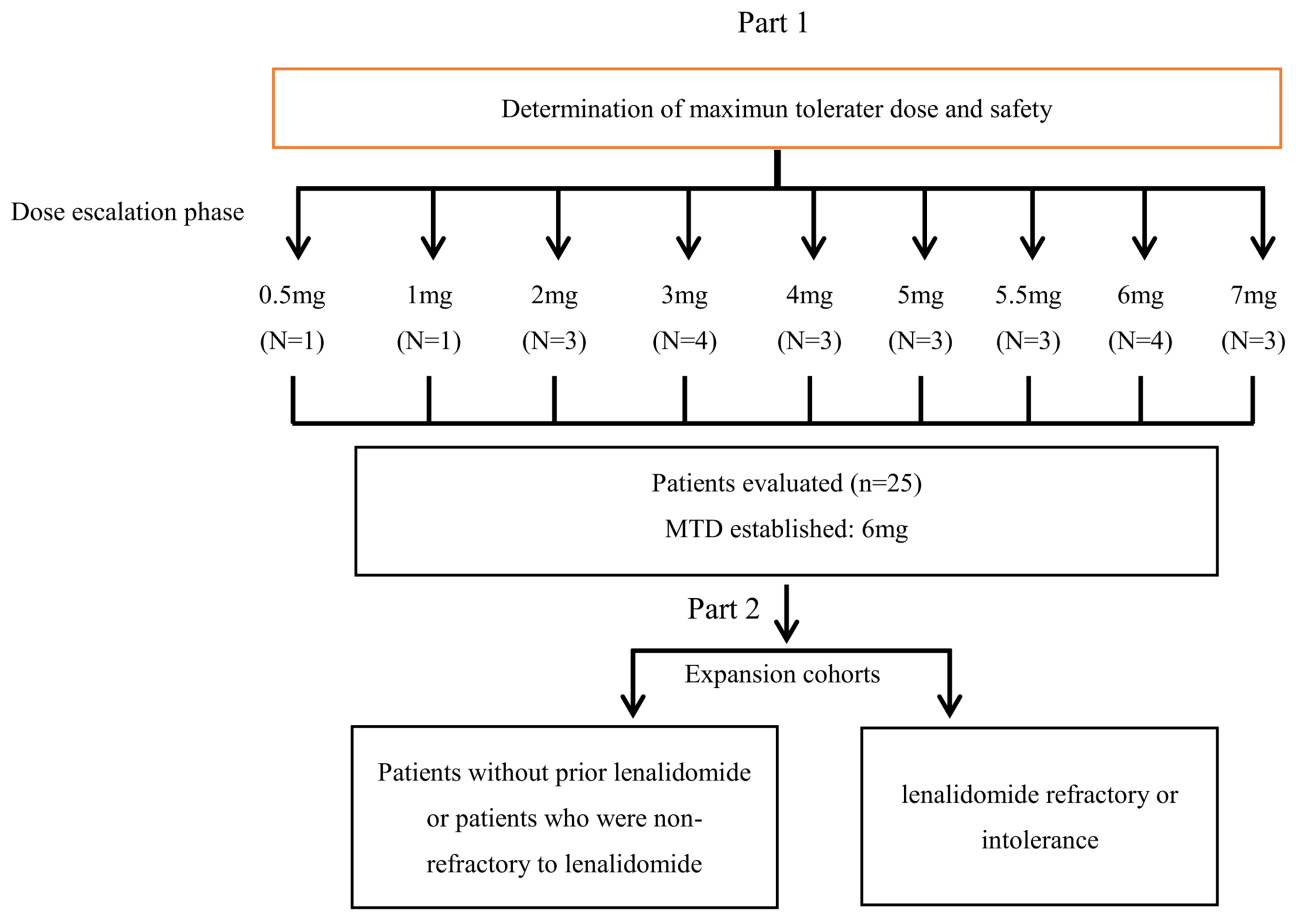
Flow chart.

This trial included dose‐escalation and dose‐expansion phases (Figure 1). Considering the results of the 28‐day repeated‐dose toxicology assay for TQB3602, the highest no‐serious toxicity dose (HNSTD) was 1.5 mg/kg for rats and 0.2 mg/kg for beagles, leading to a maximum recommended starting dose of 1.8 mg (calculated from HNSTD of rats) and 1.2 mg (calculated from HNSTD of beagles).^14^, ^15^ Eventually, the starting dose of TQB3602 for a phase I dose‐escalation trial was determined to be 0.5 mg, taking into account the clinical practice and formulation specifications, as well as the fact that the sciatic nerve injury in a beagle had not recovered. The dose selected for the dose‐expansion phase was the maximum tolerated dose (MTD) determined during the dose‐escalation phase.

The dose‐escalation phase used the standard cohort 3 + 3 design, and doses were not escalated in any individual participant. TQB3602 was taken orally with a starting dose of 0.5 mg in one or two participants and 1.0 mg in one or two participants. The subsequent enrollment of three participants in each dose group was performed in 1.0‐mg increments until the MTD was determined or until the expected efficacy dose if no MTD was observed. Alternative dosing might also be considered for dose escalation, depending on the pharmacokinetics and tolerability of TQB3602. TQB3602 was administered on days 1, 8, and 15 of each cycle, allowing for delayed dosing and dose adjustments of TQB3602 (Table S2). The dose‐limiting toxicity (DLT) observation period was the first cycle of the dose‐escalation trial, during which no dosing schedule adjustments were permitted. If no DLT or other serious adverse event (SAE) related to TQB3602 occurred during the first cycle, the treatment would continue. TQB3602 was prescribed until disease progression or unacceptable toxicity.

### 2.3 Endpoints

The primary endpoints of this study were the safety (including DLT and MTD) and the pharmacokinetics of TQB3602. DLT was defined as (1) grade ≥3 non‐hematological toxicity, except grade 3 arthralgia, myalgia, and grade 3 fatigue, herpes zoster infection, and alopecia lasting <7 days, (2) grade ≥3 vomiting, diarrhea after supportive care for 5 days, (3) grade ≥3 peripheral neuropathy or grade 2 peripheral neuropathy with pain, (4) grade ≥4 hematologic toxicity, except grade 4 neutropenia and thrombocytopenia lasting <7 days, (5) platelet count <10 × 10^9^/L, (6) grade 3 thrombocytopenia with bleeding, (7) grade 3 neutropenia with fever (body temperature ≥38.5°C) or infection, or (8) any grade toxicity that the investigator determined that the participant should terminate the study. The assessment of peripheral neuropathy adhered to the standards by CTCAE 5.0, and incorporated patient‐reported outcomes encompassing subjective symptoms reported by patients, as well as a thorough evaluation of instrumental activities of daily living (ADL) and self‐care ADL. The MTD was defined as the highest dose level at which DLT occurred in less than one‐third of participants (i.e., at least two of a maximum of six participants). AEs were assessed according to NCI‐CTCAE 5.0. After the first cycle, safety was followed up every 2 weeks in cycle 2 and 3, and then on each day 28 of the subsequent cycles. Safety follow‐up ended 28 days after the last dose of the study drug.

The pharmacokinetics/pharmacodynamics parameters included the time to peak concentration (*T*_max_), the peak concentration (*C*_max_), the area under the concentration curve (AUC_last_), etc., which were assessed 0–30 min before TQB3602 administration on days 1, 8, and 15 of the first cycle and day 1 of the second cycle, as well as 0.25, 0.5, 1, 1.5, 2, 4, 8, 12, 24, 48, 96, 120, and 144 h after TQB3602 administration on days 1 and 15 of the first cycle. These time points were selected based on the pharmacokinetics characteristics of ixazomib, which had a *T*_max_ of approximately 1–1.5 h and a half‐life of approximately 140–150 h.^16^, ^17^ These time points allowed for precise multiple assessments before reaching peak concentrations and a complete evaluation of the drug's half‐life, ensuring a thorough understanding of its pharmacokinetic profile.

The secondary endpoints included the objective response rate (ORR, i.e., the proportion of participants with complete response [CR] and partial response [PR]), clinical benefit rate (CBR, the proportion of participants with CR, PR, or minimal response [MR]), and disease control rate (DCR, the proportion of participants with CR, PR, MR or stable disease [SD]). Efficacy evaluation started from the second cycle and was carried out on day 28 ± 3 of each even‐numbered cycle, including serum protein electrophoresis, urine M protein electrophoresis, serum immunofixation electrophoresis, urine immunofixation electrophoresis, immunoglobulin quantification, and imaging examinations (every 12 weeks), bone marrow examinations (every 12 weeks), and other tests as needed by the investigator. Efficacy evaluation was performed according to the 2016 International Myeloma Working Group (IMWG) criteria.^18^

### 2.4 Statistical analysis

The safety analysis set was defined as all participants who received TQB3602 and underwent at least one safety assessment. The pharmacokinetics analysis set was defined as all TQB3602‐treated participants who received at least one pharmacokinetics sample with sufficient concentration‐time data to estimate pharmacokinetic parameters.

The SAS 9.4 software (SAS Inc., NCSU, USA) was used for statistical analysis. Phoenix WinNonlin 8.0 (Certara, Princeton, NJ, USA) was used to perform pharmacokinetics/pharmacodynamics non‐compartmental pharmacokinetic analysis. Continuous data were presented in the form of “mean ± standard deviation” or “median (range).” The categorical data are presented as “frequency (percentage).” All results in this study were presented using descriptive statistics.

## 3 RESULTS

### 3.1 Baseline characteristics

This study only reported the results of the dose‐escalation phase, in which a total of 25 participants with RRMM were included (Figure 1): one with 0.5 mg, one with 1 mg, three with 2 mg, four with 3 mg, three with 4 mg, three with 5 mg, three with 5.5 mg dose, four with 6 mg, and three with 7 mg (Figure 1). The median age of the participants was 65 (range: 37–73) years, and 16 (64%) were male. All were Asians. ECOG PS was 0 in three participants (12%), 1 in 18 (72%), and 2 in four (16%). The Revised International Staging System (R‐ISS) stage was unknown in seven participants; among the others, the R‐ISS stage was I in five participants (27.78%), II in six (33.33%), and III in seven (38.89%). The MM subtype was IgG in 15 participants (60%), IgA in eight (32%), and λ light chain in two (8%) (no κ light chain cases). Anemia was seen in one participant (4%), and bone involvement was seen in 22 (88%). The median number of prior lines of therapy was 5 (range: 2–14). Prior therapy included ixazomib (4%), bortezomib (80%), lebalidomide (80%), and thalidomide (40%). There were 13 (52%) participants refractory to bortezomib and 16 (64%) refractory to lenalidomide/thalidomide (Table 1).

**TABLE 1:** Baseline characteristics.

Characteristic	Total (*n* = 25)
Median age, years (range)	65 (37–73)
Male, *n* (%)	16 (64.00)
ECOG performance status, *n* (%)	
0	3 (12.00)
1	18 (72.00)
2	4 (16.00)
R‐ISS disease stage, *n* (%)	
I	5 (27.78)
II	6 (33.33)
III	7 (38.89)
Unknown	7
MM subtype, *n* (%)	
IgG	15 (60.00)
IgA	8 (32.00)
λ light chain	2 (8.00)
κ light chain	0
Median creatinine clearance, mL/min (range)	68.82 (33.24–127.59)
Creatinine clearance, <50 mL/min, *n* (%)	5 (20.00)
Anemia, *n* (%)	1 (4.00)
Bone disease, *n* (%)	22 (88.00)
Median β2‐microglobulin, mg/L (range)	3.08 (1.96–7.64)
Median time since MM diagnosis, years (range)	3.3 (1.2–7.7)
Median number of prior lines of therapy (range)	5 (2–14)
Prior therapy, *n* (%)	
Ixazomib	1 (4.00)
Bortezomib	20 (80.00)
Lenalidomide	20 (80.00)
Thalidomide	10 (40.00)
Refractory to last prior therapy, *n* (%)	
Bortezomib‐refractory	13 (52.00%)
Lenalidomide/thalidomide‐refractory	16 (64.00%)

Abbreviation: MM: multiple myeloma.

The majority of participants previously received bortezomib (80%) or lenalidomide (80%), while 10 (40%) participants previously received thalidomide, and one (4%) previously received ixazomib. Thirteen (52%) and 16 (64%) participants were bortezomib‐refractory and lenalidomide/thalidomide‐refractory, respectively (Table 1).

**FIGURE 2:**
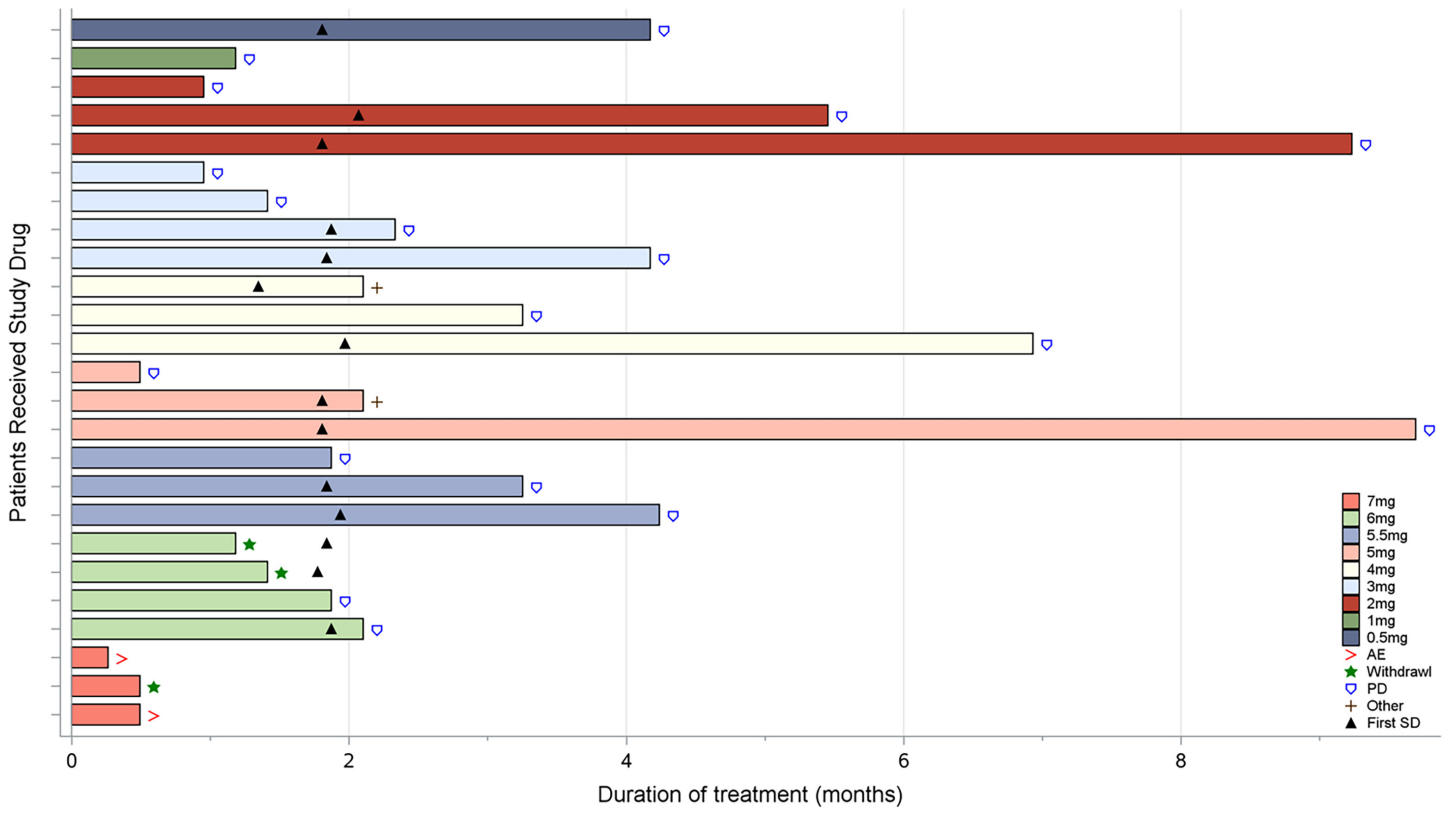
Treatment duration.

At the cutoff time of February 21, 2022, all participants had stopped treatment. Of them, three (12%) participants withdrew informed consent, 18 (72%) had disease progression, two (8%) due to adverse events (AEs), and two (8%) due to other reasons (Figure 2).

### 3.2 Safety

Two participants in the 7‐mg dose group developed DLT, one with grade 2 peripheral neuropathy complicated by pain and one with diarrhea and abdominal pain. Meanwhile, all participants in the 5.5‐ and 6.0‐mg dose groups had no DLT. Therefore, the MTD was determined to be 6.0 mg.

**FIGURE 3:**
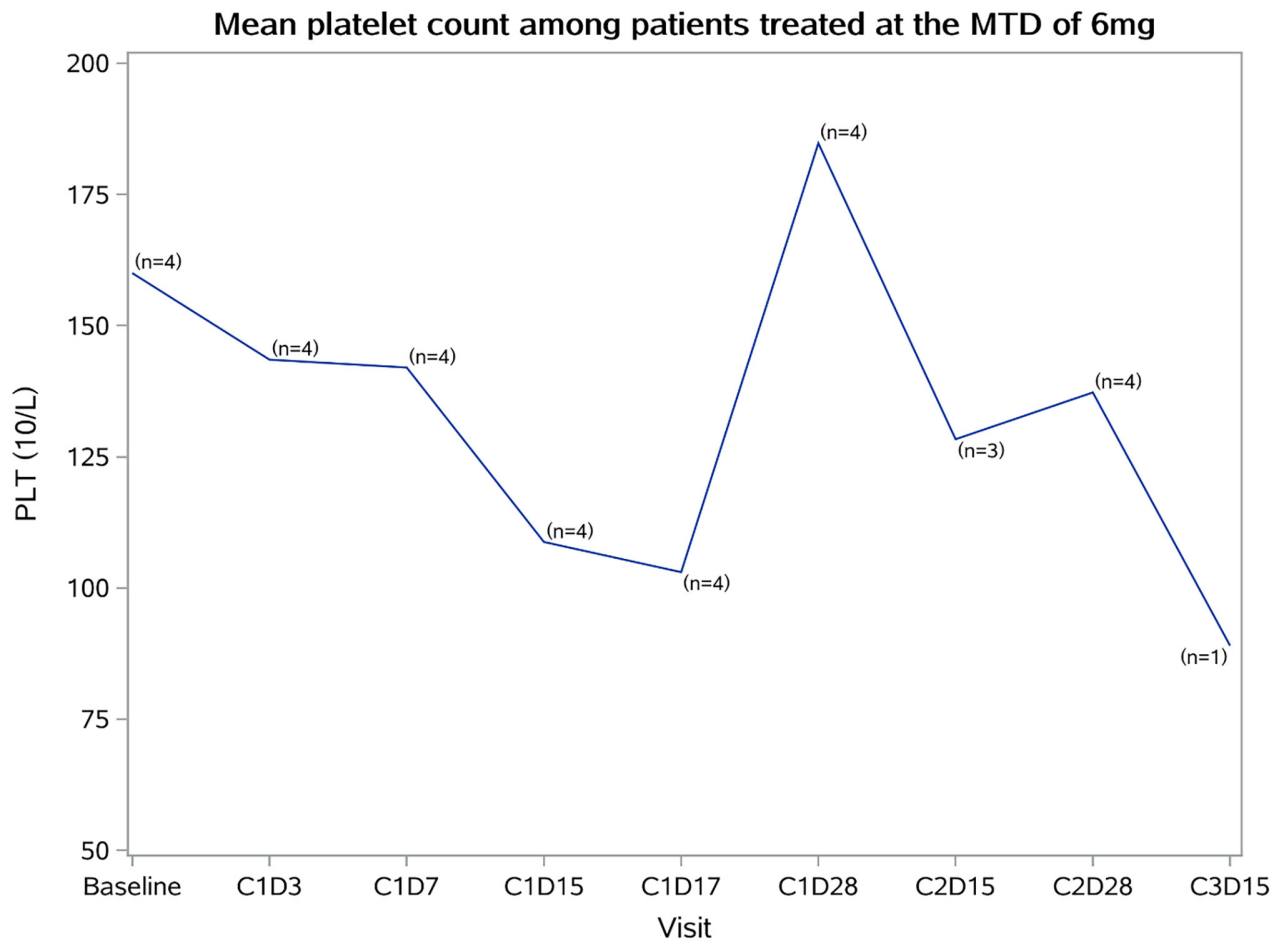
Mean platelet counts among participants treated at the maximum tolerated dose (MTD) of 6 mg.

Any‐grade AEs were reported in 96% of the participants, while grade ≥3 AEs were reported among 13 (52%) participants (Table 2). The most common AEs of all grades included thrombocytopenia (17, 68%), leukopenia (13, 52%), neutropenia (13, 52%), and anemia (12, 48%), while the most common grade ≥3 AEs anemia (6, 24%). The incidence rate of peripheral neuropathy was 16%, with no grade ≥3 peripheral neuropathy occurring. Platelet count decreased with each dose during a cycle, reaching a nadir by approximately day 17, with recovery by the planned beginning of the subsequent cycle. The platelet levels in the 6‐mg dose group are shown in Figure 3.

**TABLE 2:** Adverse events.

AEs	Total (*n* = 25)	
	All grades	Grade ≥3
Any	24 (96.0)	13 (52.0)
Hematological AEs		
Thrombocytopenia	17 (68.0)	2 (8.0)
Leukopenia	13 (52.0)	2 (8.0)
Neutropenia	13 (52.0)	3 (12.0)
Anemia	12 (48.0)	6 (24.0)
Lymphocytopenia	5 (20.0)	2 (8.0)
Non‐hematological AEs		
Diarrhea	10 (40.0)	3 (12.0)
Hypokalemia	6 (24.0)	4 (16.0)
Loss of appetite	6 (24.0)	0
Hyperglycemia	6 (24.0)	0
Positive occult blood	6 (24.0)	0
Fatigue	5 (20.0)	1 (4.0)
Elevated AST	4 (16.0)	0
Nausea	4 (16.0)	0
Vomiting	4 (16.0)	0
Peripheral neuropathy	4 (16.0)	0

Abbreviations: AEs, adverse events; AST, aspartate aminotransferase.

Severe AEs (SAEs) occurred in seven (28%) participants. Among them, there were one case of herpes zoster, one of gastrointestinal bleeding, one of intestinal obstruction, one of hypokalemia, and one of diarrhea, all possibly related to TQB3602. There were one case of anemia and one case of hypercalcemia, both unrelated to TQB3602. Except for intestinal obstruction, all AEs improved after symptomatic treatment or drug suspension.

### 3.3 Pharmacokinetics parameters

**FIGURE 4:**
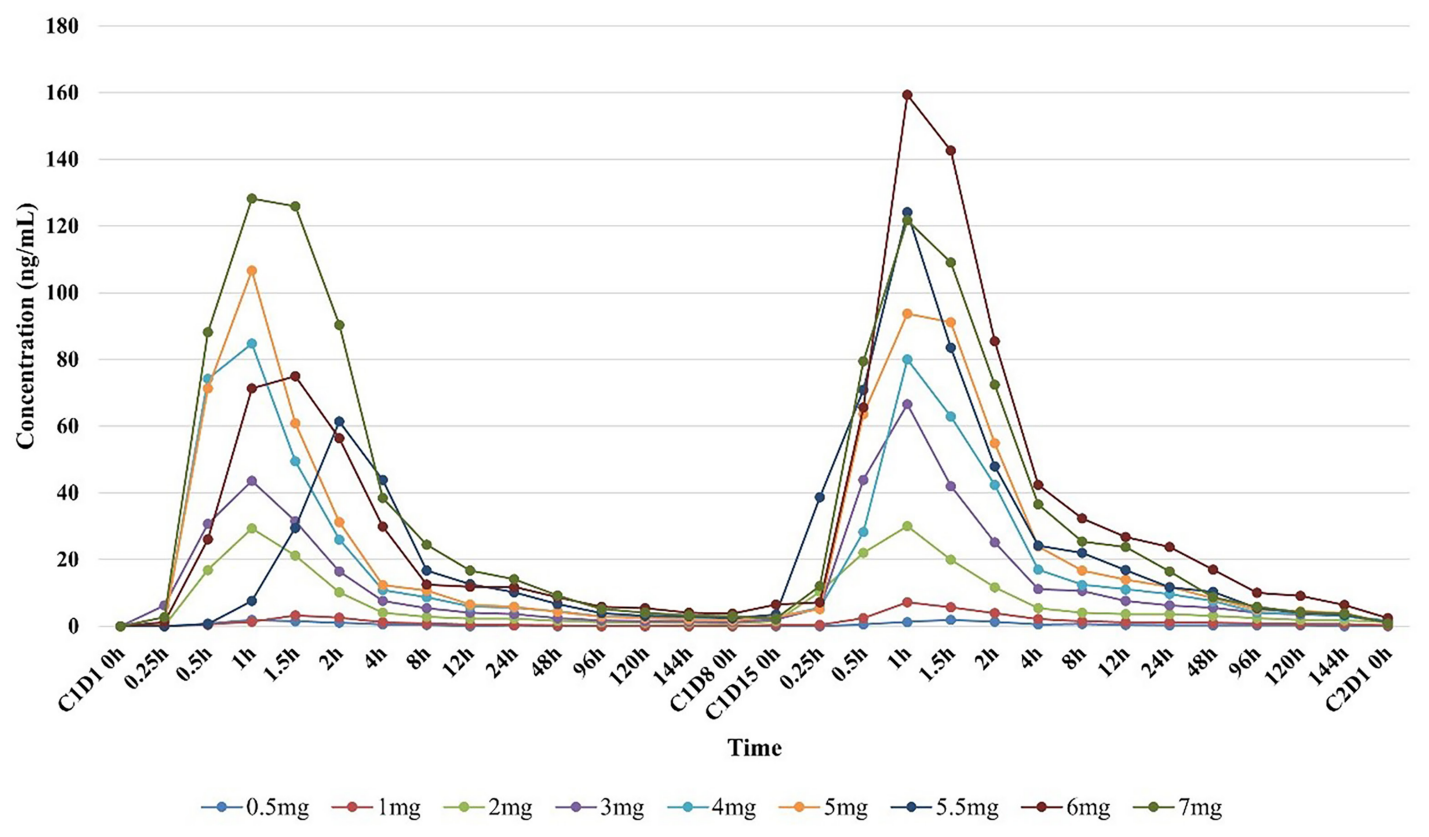
Mean plasma concentration‐time profiles of TQB3602 on days 1 and 15.

The mean plasma concentration‐time profiles of TQB3602 peaked at 1 h (range, 0.25–4.0 h), indicating that TQB3602 was rapidly absorbed, increasing the plasma concentration, and the dose increase showed an increasing linear trend (Figure 4). Furthermore, following single and multiple oral doses of TQB3602, *T*_max_ ranged from 0.8 to 2.0 h on day 1 and 0.8 to 1.5 h on day 15 (Table 3). At the 6‐mg dose level, the AUC_last_ and *C*_max_ after multiple doses were approximately 1.9 times those after a single dose. As the dose increases, the *t*_1/2_ tends to be prolonged. At a dose of 6 mg, the average *t*_1/2_ was about 79.8 h.

**TABLE 3:** Pharmacokinetic parameters on days 1 and 15.

Dose, mg	AUC_last_, h × ng/mL	*T*_max_, h	*C*_max_, ng/mL	*t*_1/2_, h
Day 1				
0.5	7.30	1	1.81	23.2
1	23.5	1.5	3.27	41.5
2	262.0	1.0	29.3	87.9
3	409.1	1.1	44.7	92.7
4	691.5	0.8	91.5	152.5
5	724.8	0.8	106.6	84.4
5.5	1058.4	2.0	61.4	64.2
6	1127.2	1.4	94.5	79.8
7	1524.9	1.2	142.9	62.2

Dose, mg	AUC_last_, h × ng/mL	*T*_max_, h	*C*_max_, ng/mL	*C*_min_, ng/mL
Day 15				
0.5	34.4	1.5	1.86	0
1	139	1	7.2	0.373
2	439.5	0.8	31.7	1.3
3	817.2	1.0	66.5	2.0
4	1040.8	0.8	81.3	2.5
5	1274.8	1.0	103.4	2.8
5.5	1403.5	0.8	146	3
6	2099.4	1	164.0	4.8
7	2399.3	1.0	182.5	3.3

*Note*: All data were presented as geometric mean.

Abbreviation: AUC, area under the concentration curve.

### 3.4 Efficacy

Efficacy was evaluable in 22 participants. Of them, 14 participants achieved SD, leading to a DCR of 63.7%. The serum M protein of two participants in the 6‐mg dose group decreased by 17% and 32%, respectively.

## 4 DISCUSSION

Bortezomib is the most commonly used proteasome inhibitor as the frontline treatment for MM, but there are still patients who inevitably develop resistance. Other proteasome inhibitors such as carfilzomib and ixazomib have been approved for RRMM by FDA. Considering the peripheral neuropathy and gastrointestinal adverse effects with ixazomib treatment,^11^ which could be caused by off‐target effects,^12^ developing a novel proteasome inhibitor with a broader safety window by eliminating off‐target effects is necessary to improve the management of patients with RRMM. To meet the need for new proteasome inhibitors with improved efficacy and tolerability, this phase I clinical trial evaluated the preliminary safety, tolerability, pharmacokinetics, and effectiveness of TQB3602, an oral proteasome inhibitor, in patients with RRMM. The MTD of TQB3602 was 6.0 mg. The incidence rates of AEs and grade ≥3 AEs were 96% and 52%, respectively. The most common AEs of all grades were mostly hematologic. The rate of peripheral neuropathy was 16%, and no grade ≥3 peripheral neuropathy occurred. TQB3602 monotherapy showed promising therapeutic effects with a disease control rate (DCR) of 63.7%. This study demonstrated the tolerability and safety of TQB3602 and preliminarily reported the efficacy potential of TQB3602. Oral agents are preferable options for frail older adult patients with MM in order to reduce visits to the clinic and improve adherence. This patient population in this study represents a cohort of individuals who have undergone extensive prior treatments with suboptimal responses and limited viable therapeutic options. The present study demonstrated that TQB3602 might be an additional treatment option for such patients undergoing later‐line treatments. Yet, it is acknowledged that the representativeness might be limited for patients outside the specific inclusion criteria due to a lack of more evidence, and the extrapolation of results to the broader RRMM population necessitates subsequent phase III clinical trials and real‐world studies postdrug approval for validation.

In the preclinical efficacy study, TQB3602 had an inhibitory effect on the 20S proteasome and tumor cell proliferation, and the initial dose of the tumor suppressor was minimal.^13^ It significantly prolonged the survival time of mice with myeloma compared with ixazomib. Therefore, TQB3602 is expected to be a proteasome inhibitor for treating RRMM with better efficacy and safety than the previous molecules available. In this phase I trial, the DLT events of TQB3602, including peripheral neuropathy and gastrointestinal toxicity, were similar to ixazomib. In spite that patients in this study were older (mean age: 65 vs. 64 years), with more advanced disease (R‐ISS disease stage III: 38.89% vs. 20%) and more heavily treated (median prior treatment lines: 5 vs. 4),^19^ TQB3602 was marginally better than ixazomib regarding neurological toxicity, with a slightly numerically lower incidence of peripheral neuropathy (16% vs. 20%), but a similar incidence of diarrhea than ixazomib (40% vs. 38%). AEs often accompany a detriment to the quality of life of patients, and it is noteworthy that in this study, the incidence of diarrhea and peripheral neuropathy was relatively low. This suggests a potential benefit to patient well‐being and treatment adherence. Both diarrhea and neurotoxicity can lead to a decline in quality of life. As the grade of AEs escalates, so does its impact on the quality of life. Moreover, these AEs also necessitate hydration therapy.^20^ The occurrence of such AEs will affect the quality of life and the activities of daily living.^21^, ^22^ Therefore, a low incidence of AEs could be potentially helpful to enhance the quality of life for patients, and subsequently, medication adherence. The incidence of grade ≥3 diarrhea was numerically lower than with ixazomib (12% vs. 17%), while the rate of grade ≥3 AEs was comparable to that of ixazomib (52% vs. 53%).^19^ Similarly, delanzomib, a novel proteasome inhibitor, may result in a numerically higher incidence of grade ≥3 diarrhea (19% vs. 12%) and peripheral neuropathy (21% vs. 16%) than TQB3602.^23^ Peripheral neuropathy is a challenge for anti‐myeloma treatment in elderly patient especially those with diabetes, and diarrhea might negatively impact the persistence of oral anti‐myeloma treatment. With a relatively high incidence of peripheral neuropathy (35%, ≥grade 3: 3%)^24^ and diarrhea (26.2%, ≥grade 3: 13.9%)^25^ reported in real‐world settings with ixazomib, TQB3602 might be a safer option for MM. Hence, the main advantage of TQB3602 compared with other proteasome inhibitors could be fewer off‐target effects and a lower incidence of peripheral neuropathy. It will have to be examined more closely in future studies.

Notwithstanding, hematologic toxicities, such as decreased platelet counts, were higher than with ixazomib (68% vs. 43%),^19^ suggesting that follow‐up studies should focus on hematological toxicity. Indeed, thrombocytopenia can increase the risk of hemorrhagic events, and managing thrombocytopenia can lead to treatment delays or interruptions, which can affect the oncological outcomes.^26^ Moreover, the transient drug‐related effects on participants' platelet count dropping after TQB3602 dosing are comparable to those reported with ixazomib.^19^ In this study, 8% of the participants discontinued treatment due to AEs, compared with 12% who discontinued ixazomib due to AEs in a study by Kumar et al.^19^ Furthermore, a low risk of neurotoxicity (16% any grade and 0% grade ≥3) was observed with TQB3602, comparable to other proteasome inhibitors with grade 1 or 2 AEs.^19^, ^23^ The other AEs included hypokalemia, loss of appetite, hyperglycemia, occult blood in the feces, fatigue, elevated liver enzymes, nausea, and vomiting. A similar AE profile was observed with ixazomib^11^, ^19^ and marizomib,^27^ except that skin toxicity and dehydration were observed with ixazomib but not with TQB3602. The common AEs of carfilzomib mostly include fatigue, anemia, nausea, thrombocytopenia, anemia, lymphopenia, and pneumonia.^28^ Nearly all patients experience diarrhea with oprozomib, while the rates of nausea, vomiting, neutropenia, anemia, and thrombocytopenia were relatively high.^29^ The finding of this phase I clinical trial was consistent with the in vitro and in vivo toxicological studies that demonstrated that TQB3602 has a good toxicity profile and its toxic effects were comparable to those of ixazomib and other proteasome inhibitors. Besides thrombocytopenia, the trial also observed cases of leukopenia, neutropenia, and anemia, which are common hematological toxicities of cancer treatments.^30^ Leukopenia and neutropenia can increase the risk of infections,^31^ and anemia can influence survival.^32^ Future studies will have to examine closely the hematological toxicities of TQB3602.

After the participants received oral TQB3602, the *T*_max_ was 0.8–1.5 h, suggesting a fast absorption in humans. With the increase in drug dose, AUC and *C*_max_ also showed an increasing trend, and the increase ratio was linear with the dose increase ratio. A higher *C*_max_ with increasing doses would indicate a higher availability and action of the drug with higher doses. Intra‐individual *t*_1/2_ varied greatly, ranging from 23 to 152 h. At the 6‐mg dose level, the mean *t*_1/2_ was approximately 80 h, and AUC_last_ and *C*_max_ were approximately 1.9 times higher after multiple doses than after a single dose. Within the explored dose range, absorption of TQB3602 might not have reached saturation levels, especially after multiple doses at day 15. Consequently, higher doses are associated with increased peak drug concentrations and exposure, suggesting a potential correlation with improved therapeutic efficacy. However, the occurrence of DLTs in the 7 mg dose group prompted the determination of the MTD at 6 mg. Thus, we have identified 6 mg as the recommended dose for subsequent studies. This decision on dose ensures that it might achieve the highest expected therapeutic benefits while maintaining safety within the investigated range. Moreover, based on the stable and predictable pharmacokinetic profile observed with the current dosing regimen (oral administration on days 1, 8, and 15 of each cycle), it is suggested that this schedule be continued in future investigations. This approach aims to optimize drug efficacy while providing a consistent and manageable drug metabolism profile. Lastly, it should be noted that the elimination mechanisms of TQB3602 are still mostly unknown. Drug elimination mostly depends upon liver metabolism and kidney excretion.^33^ Whether polymorphisms in cytochrome enzymes, liver function, or kidney function can influence the *t*_1/2_ of TQB3602 remains to be explored.

The efficacy results showed that the M protein of the participants decreased at the MTD dose and that the DCR was 63.7%. The change in M protein after treatments is a prognostic indicator in MM,^34^ and a decrease in the M protein observed here supports the observed DCR. The antitumor activity of TQB3602 monotherapy was better than ixazomib (DCR: 50%),^19^ delanzomib (SD rate: 55%),^23^ and carfilzomib (DCR: 39%) in a phase I trial.^35^ Since proteasome inhibitors are often combined with other drugs, the preliminary single‐drug antitumor effects evaluated in the dose‐escalation phase of this phase I clinical trial are limited for reference only. The prognostic value of the M protein in the context of TQB3602 treatment warrants further investigation in future studies. The expansion cohort will explore the efficacy of further combination therapy with pomalidomide and glucocorticoids, since combination therapy stands as a well‐established and effective strategy, particularly for the management of relapsed and refractory disease. In a subcutaneous xenograft mouse model of human myeloma MM.1S cells, preclinical data showed that TQB3602 had a significant antitumor effect alone and even more when used in combination with lenalidomide. Furthermore, previous clinical trials have examined the combination of protease inhibitors with pomalidomide and dexamethasone.^36^, ^37^, ^38^ Considering these clinical experiences and existing evidence, we are encouraged to explore the potential synergies of TQB3602 with pomalidomide and dexamethasone in our ongoing trial.

The relationship between autophagy, sensitivity to protease inhibitors, and resistance in MM is intricate, complex, and context‐dependent. Autophagy can enhance bortezomib sensitivity by clearing misfolded proteins induced by proteasome inhibition, but it can also act as a protective mechanism that contributes to bortezomib resistance by promoting cell survival under stress conditions.^39^, ^40^ Clinical implications include the development of dual‐targeting strategies to inhibit the proteasome and modulate autophagy simultaneously.^41^ Future studies will examine biomarkers that could help predict patient response, guide personalized treatment, and examine the interplay between proteasome inhibition and autophagy to improve treatment strategies.

This study has limitations. The sample size of 25 was relatively small in this study, which might potentially limit the robustness of the findings regarding rare adverse events or specific patient subgroups. The study was conducted exclusively in China, limiting the generalizability of the findings to a broader population. The patient selection criteria, which included patients aged 18–75 years old and those received ≥2 prior antimyeloma therapies, might restrict the generalizability of the study conclusion to younger or older patients or those with less treatment exposure. Besides, this study also excluded patients with peripheral neuropathy of grade 2 or higher, which may have inadvertently excluded a subgroup of patients who are more prone to neurotoxicity. Therefore, future studies are warranted to include diverse patient populations across multiple geographic locations and with varying characteristics such as age ranges, treatment settings and peripheral neuropathy to enhance the external validity of these findings. On the other hand, the long‐term safety and efficacy of TQB3602‐based combinations could be further explored in future large‐scale studies. Finally, predefined outcomes of the dose‐escalation part have been reported in the manuscript, while the dose‐expansion results are not yet mature and will be reported later.

In conclusion, TQB3602 in this phase I dose‐escalation trial exhibited favorable safety and tolerability in RRMM with the identified MTD of 6 mg. TQB3602 monotherapy shows an encouraging antitumor effect. The safety and efficacy of the combination of TQB3602, pomalidomide, and dexamethasone will be further investigated in the ongoing dose‐expansion trial (NCT04275583).

## AUTHOR CONTRIBUTIONS

**Wenjiao Tang:** Conceptualization (equal); investigation (equal); writing – original draft (equal); writing – review and editing (equal). **Yan Li:** Investigation (equal). **Li Zhang:** Investigation (equal); resources (equal). **Xushu Zhong:** Investigation (equal). **Qiushi Liang:** Investigation (equal). **Yuhuan Zheng:** Investigation (equal). **Yuzhang Liu:** Investigation (equal). **Yafei Wang:** Conceptualization (equal). **Xunqiang Wang:** Conceptualization (equal); project administration (equal). **Yun Zeng:** Investigation (equal). **Baijun Fang:** Investigation (equal). **Li Zheng:** Conceptualization (equal). **Ting Niu:** Conceptualization (equal); formal analysis (equal); writing – review and editing (equal).

## CONFLICT OF INTEREST STATEMENT

Xunqiang Wang and Yafei Wang are employees of Chia Tai Tianqing Pharmaceutical Group Co., Ltd. The remaining authors declare that the research was conducted in the absence of any commercial or financial relationships that could be construed as a potential conflict of interest.

## ETHICS STATEMENT

This study was approved by the Ethics Review Board of West China Hospital, Sichuan University (2019 clinical trial [Western Medicine] approval [243]). In accordance with the Declaration of Helsinki, written informed consent was obtained from all participants before enrollment. The trial was registered with ClinicalTrials.gov (NCT04275583).

## Supporting information


Table S1.

## Data Availability

All data generated or analyzed during this study are included in this published article and its supplementary information files.
