# Supplementary material for: Phase I study of TQB3602, an oral proteasome inhibitor, in relapsed and refractory multiple myeloma
Source: Cancer Med. 2024 Jul 19;13(14):e7435. doi: 10.1002/cam4.7435 (PMC11259557; doi:10.1002/cam4.7435)
Supplement: Supplementary file 1 — Table S1. [file CAM4-13-e7435-s001.docx]

***Supplementary materials***

**Phase I study of TQB3602, an oral proteasome inhibitor, in relapsed and refractory multiple myeloma**

**Supplementary Table 1.** Inclusion and exclusion criteria

| **Inclusion criteria**   1. Voluntarily joined the study and signed the informed consent; 2. Age 18~75 years old (including the threshold value), both male and female; 3. Eastern Cooperative Oncology Group (ECOG) score: 0, 1, or 2; 4. Expected survival of more than 12 weeks; 5. MM patients who relapsed or failed after previous ≥2 lines of therapies (including lenalidomide or thalidomide, bortezomib, and glucocorticoids) and have received or were ineligible for hematopoietic stem cell transplantation (HSCT); 6. Patients with relapsed or refractory MM must have at least one measurable lesion: serum M protein ≥ 5 g/L or urine M protein ≥ 200 mg/24 h. Only serum free light chain level as measurable lesions: serum free light chain level ≥ 10 mg/dL and serum free light chain ratio κ/λ ratio abnormal; 7. Major organ function within 14 days before study drug administration, meeting the following criteria: 8. Routine blood examination standards (without receiving a blood transfusion, erythropoietin, granulocyte colony-stimulating factor, or other medical support within 14 days before the examination): absolute neutrophil value ≥ 1.0×109/L, platelet count ≥ 75×109/L, hemoglobin ≥ 75g/L; 9. Alanine aminotransferase (ALT) and aspartate aminotransferase (AST) ≤ 2.5 × upper limit of normal (ULN), total bilirubin ≤ 2.0 × ULN; 10. Creatinine clearance ≥ 30 mL/min; 11. Corrected serum calcium ≤ ULN; 12. Cardiac function: Echocardiographic assessment of left ventricular ejection fraction (LVEF) ≥ 50%; 13. Males and females of childbearing potential of childbearing potential must agree to use effective contraception from the time of signing the informed consent form until 180 days after the last dose of the study drug. Females of childbearing age include premenopausal females and females within 2 years after menopause. Females of childbearing potential must have a negative blood pregnancy test result within ≤ 7 days before the first dose of the study drug. |
| --- |
| **Exclusion criteria:**   1. Allergic constitution, or known allergy to the research drugs or excipients; 2. Patients with peripheral neuropathy of grade ≥ 2; or patients with grade 1 peripheral neuropathy with pain; 3. Patients with diarrhea > 1 grade during the screening period; (Grade 1 diarrhea: compared with baseline, the number of stools increased < 4 times per day; the discharge from the ostomy was slightly increased.) 4. Has received chemotherapy, radiotherapy, targeted therapy, immunotherapy, or other systemic antitumor therapy within 14 days before taking the study drug for the first time; 5. Patients who have received ixazomib treatment before taking the study drug for the first time and have less than 5 half-lives in the ixazomib elution period; 6. Previously received allogeneic stem cell transplantation; 7. Has received autologous stem cell transplantation within 12 weeks before enrollment; 8. Suffering from any acute or chronic gastrointestinal diseases or received gastrointestinal medical operations and other factors that may affect the absorption, distribution, metabolism, excretion, and tolerance evaluation of the study drug (such as inability to swallow drugs after gastrointestinal resection, persistent diarrhea, and intestinal obstruction, etc.); 9. Clinically symptomatic brain metastases, spinal cord compression, cancerous meningitis, or other evidence of uncontrolled metastases to the patient’s brain or spinal cord, or known active central nervous system (CNS) involvement or evidence of MM meningeal involvement of clinical signs, and patient judged by the investigator to be unsuitable for enrollment; 10. Pregnant and breastfeeding female 11. Evidence of severe or uncontrolled systemic disease (e.g., unstable or decompensated respiratory disease, liver disease, or kidney disease) or those who have undergone major surgery; 12. Patients with hypertension (systolic blood pressure ≥ 140 mmHg, diastolic blood pressure ≥ 90 mmHg) who were still poorly controlled after drug treatment, past or current heart failure, myocardial ischemia, or myocardial infarction, unstable angina pectoris, arrhythmia (including QTc interval: male > 450 ms, female > 470 ms); 13. Suffering from active or a history of immunodeficiency, including HIV test positive or suffering from other acquired or congenital immunodeficiency diseases, or a history of organ transplantation; 14. Patients with a history of other serious underlying diseases, such as:   a. History of a clear neurological or psychiatric disorder, including epilepsy or dementia.  b. HBsAg positive and peripheral blood hepatitis B virus deoxyribonucleic acid (HBV DNA) test is higher than the upper limit of normal, HCV antibody positive, or syphilis test positive.  c. There was an infection requiring systemic treatment.   1. Use CYP3A4 inducer/inhibitor drugs before the first dose of the study drug and within 5 half-lives of CYP3A4 inducer/inhibitor drugs; 2. Non-hematological toxicity caused by previous antitumor therapy has not recovered to CTCAE 5.0 grade evaluation ≤ grade 1 (except for alopecia); 3. Other malignant tumors have occurred or are currently suffering from other malignant tumors within 3 years before the first medication. The following two conditions were eligible for enrollment: other malignancies treated with a single surgery, achieving 5 consecutive years of disease-free survival (DFS); cured cervical carcinoma in situ, non-melanoma skin cancer, and superficial bladder tumors [ Ta (non-invasive tumor), Tis (carcinoma in situ) and T1 (tumor-infiltrating basement membrane)]; 4. Those who have used other clinical trial drugs within 28 days before taking the study drug for the first time; 5. Investigators believed that they were not suitable to participate in clinical trials: including those with concomitant diseases or abnormal examinations that seriously endanger the safety of patients or affect the completion of the study, according to the judgment of the researchers. |

**Supplementary Table 2.** Dosage adjustment protocol

| **Recommendations for non-hematological toxicity protocol adjustment** | |
| --- | --- |
| CTCAE grade | Recommendations for adjustment |
| Grade 1/grade 2 | Continue study treatment and use at the current dose  Observe closely  Provide supportive care according to hospital standards as needed (or refer to recommended management advice) |
| Grade 3 or grade 4 | Suspend study treatment and observe closely. Provide supportive care according to hospital standards (refer to recommended management recommendations). When toxicity resolved to ≤ Grade 1 or baseline, study treatment was resumed, and the dose was reduced by one level. If grade 3 or higher toxicity recurred, the study regimen was discontinued. |
| **Recommendations for protocol adjustment in peripheral neuropathy** | |
| CTCAE grade | Recommendations for adjustment |
| Grade 1 peripheral neuropathy with pain or grade 2 peripheral neuropathy | Suspend study treatment, observe closely, and provide supportive treatment according to hospital standards if necessary (or refer to recommended treatment recommendations). When peripheral neuropathy recovers to ≤ grade 1 without pain, or returns to the patient’s baseline level, continue the original plan treatment. |
| Grade 2 peripheral neuropathy with pain or grade 3 peripheral neuropathy | Suspend study treatment, observe closely, provide supportive care according to hospital standards (or refer to recommended management recommendations), and resume study treatment with dose reduction-one level when toxicity returns to baseline conditions or ≤ grade 1 without pain. |
| Grade 4 peripheral neuropathy | Discontinue the study treatment regimen. |
| **Recommended dose adjustment for hematological toxicity** | |
| CTCAE grade | Recommendations for program adjustment |
| A platelet count below 75,000/mm^3^ | Suspend study treatment and observe closely. Provide supportive care according to hospital standards (refer to recommended management recommendations). When toxicity returned to at least 75,000/mm^3^), study treatment was resumed, and the dose remained unchanged. |
| A platelet count below 30,000/mm^3^ | Suspend study treatment and observe closely. Provide supportive care according to hospital standards (refer to recommended management recommendations). When toxicity returned to at least 75,000/mm^3^), study treatment was resumed, and one dose level was reduced. |
| Absolute neutrophils below 1000/mm^3^ | Suspend study treatment and observe closely. Provide supportive care according to hospital standards (refer to recommended management recommendations). When toxicity returned to at least 1000/mm^3^), study treatment was resumed, and the dose remained unchanged. |
| Absolute neutrophils below 500/mm^3^ | Suspend study treatment and observe closely. Provide supportive care according to hospital standards (refer to recommended management recommendations). When toxicity returned to at least 1000/mm^3^), study treatment was resumed, and one dose level was reduced. |
| Other hematological toxicity grades 3 or 4 | If clinically indicated, suspend study treatment and observe closely. If necessary, increase the frequency of blood routine examination, and provide supportive treatment according to hospital standards (or refer to recommended treatment recommendations). When toxicity resolves to ≤ grade 2 or baseline (and no infection or bleeding tendencies), study treatment can be restarted at the current dose or dose reduction-one level. |
